# Repeatability of binarization thresholding methods for optical coherence tomography angiography image quantification

**DOI:** 10.1038/s41598-020-72358-z

**Published:** 2020-09-21

**Authors:** Nihaal Mehta, Phillip X. Braun, Isaac Gendelman, A. Yasin Alibhai, Malvika Arya, Jay S. Duker, Nadia K. Waheed

**Affiliations:** 1 Department of Ophthalmology, New England Eye Center, Tufts Medical Center, 800 Washington Street, Box 450, Boston, MA 02111 USA; 2 Department of Ophthalmology, University of Colorado, Denver, CO USA; 3 Flaum Eye Institute, University of Rochester Medical Center, Rochester, NY USA; 4 Department of Surgery, Tufts Medical Center, Boston, MA USA

**Keywords:** Optical imaging, Translational research

## Abstract

Binarization is a critical step in analysis of retinal optical coherence tomography angiography (OCTA) images, but the repeatability of metrics produced from various binarization methods has not been fully assessed. This study set out to examine the repeatability of OCTA quantification metrics produced using different binarization thresholding methods, all of which have been applied in previous studies, across multiple devices and plexuses. Successive 3 × 3 mm foveal OCTA images of 13 healthy eyes were obtained on three different devices. For each image, contrast adjustments, 3 image processing techniques (linear registration, histogram normalization, and contrast-limited adaptive histogram equalization), and 11 binarization thresholding methods were independently applied. Vessel area density (VAD) and vessel length were calculated for retinal vascular images. Choriocapillaris (CC) images were quantified for VAD and flow deficit metrics. Repeatability, measured using the intra-class correlation coefficient, was inconsistent and generally not high (ICC < 0.8) across binarization thresholds, devices, and plexuses. In retinal vascular images, local thresholds tended to incorrectly binarize the foveal avascular zone as white (i.e., wrongly indicating flow). No image processing technique analyzed consistently resulted in highly repeatable metrics. Across contrast changes, retinal vascular images showed the lowest repeatability and CC images showed the highest.

## Introduction

Quantification of optical coherence tomography angiography (OCTA) images has been widely applied in recent years. Countless studies have been published analyzing quantification metrics from retinal vascular and choriocapillaris (CC) OCTA images. There are a wide range of metrics used in these studies, ranging from simple quantification of flow area to more complex vessel and non-flow area analyses. Generally, the most common metric used in OCTA analysis is vessel area density (VAD), which analyzes the proportion of white pixels in a binarized OCTA image in an attempt to quantify the amount of blood flow. Another common metric used in retinal vascular OCTA images is vessel length (VL), which totals the length of vessels in a skeletonized image. In CC OCTA images, various metrics have been applied to the “flow deficits” or “non-flow areas,” which appear black in binarized images. These include number, total area, percentage, and average size of flow deficits.

In many cases, studies use proprietary software in-built into the OCTA system in order to calculate these metrics. The Optovue Avanti RTVue XR SD-OCT (Optovue, Inc., Fremont, CA, USA), for example, can calculate several quantification metrics automatically. The Cirrus HD-OCT 5000 (Carl Zeiss Meditec, Inc., Dublin, CA, USA) can also calculate metrics, although this software capability is not currently available in all countries. Past studies suggest that the repeatability of quantification using the Avanti and Cirrus systems’ in-built analytics is relatively high, at least for some metrics^1,2^. However, in studies that use systems without quantification capabilities, these metrics need to be calculated using standalone software following image export. A critical step in this process of image quantification is creating a binarized (black-and-white) image from the grayscale OCT angiogram using a thresholding algorithm. We have previously shown that the method of binarization thresholding has a statistically significant impact on resulting quantification metrics, and that many binarization thresholding algorithms are highly susceptible to alterations in image contrast, which are often made during analysis or even on the imaging system itself before image export^3^. Rabiolo et al. also showed that variations in analysis methods can result in different VAD values, and that there is often poor correlation between methods^4^. Most recently, Chu et al. have raised some critical questions regarding the appropriateness of previously applied methods for analysis of CC OCTA images^5^. There is little consistency in the methods applied between research studies, which limits the ability to generate standard numerical metrics across different devices and techniques. Moreover, the repeatability of metrics produced using different methods, including binarization thresholds, as they have been applied in previous studies, remains an open question. It is important to note that the relevance of this question is not limited just to past and ongoing OCTA research studies, as these metrics are already being explored as endpoints in ongoing clinical trials. This underscores the importance and clinical relevance of better understanding the repeatability of these metrics.

The present study set out to measure the repeatability of metrics produced using a near-comprehensive range of binarization thresholding methods that have already been applied in previous OCTA studies. We also assessed whether contrast changes and several image processing techniques, including registration, histogram normalization, and contrast-limited adaptive histogram equalization, affect repeatability.

## Methods

The study protocol was approved by the Tufts Medical Center Institutional Review Board (IRB) and adhered to the tenets of the Declaration of Helsinki and the Health Insurance Portability and Accountability Act of 1996. Written informed consent was obtained in accordance with the Tufts Medical Center IRB.

### Image acquisition

Three successive 3 × 3 mm OCTA images of 13 healthy eyes were obtained by a trained ophthalmic photographer on each of three systems (a total of nine images per eye): the Carl Zeiss PLEX Elite 9000 (Carl Zeiss Meditec, Inc., Dublin, CA, USA), the Carl Zeiss Cirrus HD-5000, and the Optovue Avanti RTVue XR. The PLEX system images with a 1060 nm central wavelength light source and a bandwidth of 100 nm and operates at 100,000 A-scans per second, an A-scan depth of 3 mm, an axial resolution of 6.3 µm, and a transverse resolution of 20 µm. 3 × 3 mm OCTA en-face images on the PLEX are constructed of 300 A-scans per B-scan and 300 B-scans per volume. The Cirrus system images with an 840 nm central wavelength and operates at 27,000–68,000 A-scans per second, an A-scan depth of 2 mm, an axial resolution of 5 µm, and a transverse resolution of 15 µm. 3 × 3 mm OCTA en-face images are constructed on the Cirrus from 245 A-scans per B-scan and 245 B-scans per volume. The Avanti system images with an 840 nm central wavelength and operates at 70,000 A-scans per second, an A-scan depth of 2–3 mm, an axial resolution of 5 µm, and a transverse resolution of 15 µm. 3 × 3 mm OCTA en-face images are constructed on the Avanti from 304 A-scans per B-scan and 304 B-scans per volume. For all devices, en-face image slabs of the full retinal layer (FRL), superficial capillary plexus (SCP), deep capillary plexus (DCP), and choriocapillaris (CC) were generated using the default automated segmentation boundaries and exported as grayscale images. The PLEX and Cirrus default segmentation boundaries are as follows: FRL = internal limiting membrane (ILM) to 70 µm above the retinal pigment epithelium fit (RPEfit) line, SCP = ILM to inner plexiform layer (IPL), DCP = IPL to outer plexiform layer (OPL), and CC = 29 µm to 49 µm below RPEfit. The Avanti default boundaries are: FRL = ILM to 9 µm below the OPL, SCP = ILM to 9 µm below the IPL, DCP = 9 µm below the IPL to 9 µm below the OPL, and CC = 9 µm above Bruch’s membrane (BM) to 31 µm below BM.

### Image analysis

Following export, all image analysis was completed using ImageJ (v 2.0.0, National Institutes of Health, Bethesda, MD, USA), and all steps were automated using a macro. Images were first converted to 8-bit (grayscale pixel values 0–255).

The following contrast adjustments were then applied separately to each image using pointwise pixel transformations: (1) Contrast was increased by applying the following transformation (where *p* represents the original 0 to 255 pixel value, and *f*(*p*) represents the final pixel value): *f*(*p*) = 1.5(*p* − 128) + 128. (2) A larger contrast increase was applied using the following transformation: *f*(*p*) = 2.0(*p* − 128) + 128.

Several image processing techniques were also separately applied to each image: (1) Histogram normalization, which scales image histograms such that the minimum pixel value is 0 and the maximum is 255, was performed in ImageJ using the “Enhance Contrast” feature with the “Normalize” option (default parameters retained: 0.3% saturated pixels). (2) Contrast-limited adaptive histogram equalization (CLAHE) was performed in ImageJ using the “Enhance Local Contrast (CLAHE)” plug-in (default parameters retained: block size 127, histogram bins 256, maximum slope 3.00, fast processing)^6^. (3) Linear registration was performed with the ImageJ plug-in “Register Virtual Stack Slices,” which uses the scale-invariant feature transform and multi-scale oriented patches algorithms with random sample consensus for feature extraction, and a rigid registration model^7–9^.

Finally, each image (the original unaltered image as well as versions with each of the above changes) was separately binarized using the following methods, which were identified as representing a near-comprehensive list of binarization algorithms (with the exception of custom methods that could not be reproduced) used in past OCTA studies: Global default^10,11^, global Huang^12^, global IsoData^13^, global mean^14,15^, global Otsu^16^, local Bernsen^17,18^, local mean^19^, local median^12,20^, local Niblack^21–23^, local Otsu^24^, and local Phansalkar^25–28^. All local thresholds were applied with a radius of 15 pixels based on the methods used in prior studies on the same devices we employed^25–28^. This radius equates to 43.9 µm for 1024 × 1024 pixel PLEX images, 104.9 µm for 429 × 429 pixel Cirrus images, and 148.0 µm for 304 × 304 pixel Avanti images.

For quantification, vessel area density (VAD) was assessed from the binarized images in all plexuses using the “Measure” feature in ImageJ. Images of the SCP, DCP and FRL were skeletonized using the “Skeletonize” plug-in, which applies binary thinning to the image^29^, and vessel length (VL) measured from the skeletonized images using the “Analyze Skeleton” plug-in, which tags and counts all pixels in the skeletonized image^30^. Finally, the number of flow deficits and average size of flow deficits in the binarized inverted choriocapillaris images were measured using the “Analyze Particles” feature. We did not calculate vessel density index or CC flow deficit percentage; although these are commonly applied metrics, they can be calculated from the metrics here reported (eg, CC flow deficit percentage = 1 − vessel area density) and thus their inclusion would introduce co-linearity into our dataset.

### Statistical analysis

All statistical analysis was completed using Stata/SE 15.1 (StataCorp, College Station, TX, USA) and Microsoft Excel (Microsoft Corporation, Redmond, WA, USA). To quantify the repeatability of quantitative metrics across multiple acquisitions of the same eye on the same device, the intraclass correlation coefficient (ICC) was calculated. The ICC estimates the proportion of variation within a data set that is attributable to between-subject variation as opposed to within-subject variation^31^. “High” repeatability was considered to be an ICC value above 0.80 and “low” an ICC below 0.50, using previously described definitions^32^. A high ICC value indicates that most variation is between subjects, and thus a high degree of repeatability (minimal variation) in within-subject measurements. However, a low ICC could also be indicative of relatively little variation between normal subjects. To assess the repeatability of binarization thresholds, including after applying image processing techniques, a one-way random effects ICC model was applied. To compare repeatability across contrast adjustment (no change, increase by factor of 1.5, increase by factor of 2.0), a two-way mixed effects model was applied. Because three images from each eye were available, the ICCs across contrast changes were calculated for each image and averaged. On occasion, negative ICC values were generated, which is due to the way many statistical software packages calculate ICC and is not meaningful in and of itself but should be interpreted as indicating a low degree of repeatability^33^. Changes in repeatability following image processing were assessed as the differences in ICC values, with a positive difference representing improvement.

## Results

In total, 13 eyes of 7 healthy individuals were imaged. The mean age was 28.3 ± 3.6 years. Mean values and distributions of all baseline measurements are summarized in Supplementary Figs. 1–3.

### Intra-subject repeatability

The ICCs for each metric (VAD, VL, number of CC flow deficits, and average size of CC flow deficits) are shown in Tables 1, 2 and 3. ICC values were inconsistent and, in general, not high.

**Table 1:** Intraclass correlation coefficients for vessel area density on different devices and plexuses.

	Device											
	PLEX Elite				Cirrus HD-5000				Optovue Avanti			
Binarization threshold	FRL	SCP	DCP	CC	FRL	SCP	DCP	CC	FRL	SCP	DCP	CC
Global Default	0.75	0.73	0.67	0.42	0.14	0.09	0.41	0.44	0.33	0.32	0.44	0.86
Global Huang	0.59	0.48	0.72	0.23	0.24	0.23	0.58	0.42	− 0.18	0.36	0.34	0.67
Global IsoData	0.72	0.72	0.65	0.18	0.16	0.14	0.34	0.37	0.35	0.44	0.43	0.86
Global Mean	0.71	0.70	0.71	− 0.05	0.16	0.12	0.43	0.37	0.23	0.28	0.49	0.81
Global Otsu	0.73	0.68	0.71	0.23	0.24	0.11	0.41	0.47	0.31	0.29	0.40	0.84
Local Bernsen	0.63	0.59	0.48	0.46	0.06	− 0.18	− 0.18	0.47	0.21	0.11	0.22	0.80
Local Mean	0.76	0.73	0.55	0.43	0.22	0.05	0.11	0.51	0.28	0.49	0.45	0.87
Local Median	0.80	0.55	0.47	0.10	0.47	0.51	0.65	0.81	− 0.07	− 0.15	− 0.09	− 0.21
Local Niblack	0.78	0.71	0.57	0.42	0.32	0.11	0.03	0.55	0.29	0.38	0.38	0.77
Local Otsu	0.77	0.70	0.56	0.45	0.32	0.12	0.09	0.53	0.32	0.35	0.39	0.85
Local Phansalkar	0.71	0.68	0.56	0.42	0.11	0.07	0.21	0.20	0.36	0.39	0.40	0.88

*FRL* full retinal layer, *SCP* superficial capillary plexus, *DCP* deep capillary plexus, *CC* choriocapillaris.

**Table 2:** Intraclass correlation coefficients for vessel length on different devices and retinal plexuses.

	Device								
	PLEX Elite			Cirrus HD-5000			Optovue Avanti		
Binarization threshold	FRL	SCP	DCP	FRL	SCP	DCP	FRL	SCP	DCP
Global Default	0.84	0.83	0.78	0.25	0.22	0.45	0.48	0.39	0.45
Global Huang	0.91	0.87	0.88	0.45	0.44	0.63	0.54	0.63	0.21
Global IsoData	0.80	0.81	0.78	0.24	0.25	0.38	0.48	0.46	0.52
Global Mean	0.83	0.86	0.85	0.43	0.42	0.60	0.59	0.50	0.49
Global Otsu	0.80	0.80	0.82	0.30	0.22	0.44	0.42	0.34	0.40
Local Bernsen	0.69	0.70	0.49	0.22	0.04	− 0.19	0.27	0.17	0.22
Local Mean	0.79	0.82	0.59	0.42	0.44	0.31	0.58	0.60	0.57
Local Median	0.75	0.77	0.60	0.51	0.67	0.62	0.60	0.66	0.58
Local Niblack	0.78	0.81	0.62	0.43	0.37	0.22	0.50	0.52	0.46
Local Otsu	0.80	0.82	0.64	0.40	0.27	0.12	0.45	0.45	0.38
Local Phansalkar	0.82	0.82	0.57	0.24	0.18	0.22	0.49	0.48	0.56

*FRL* full retinal layer, *SCP* superficial capillary plexus, *DCP* deep capillary plexus.

**Table 3:** Intraclass correlation coefficients for number and average size of flow deficits on different devices and retinal plexuses.

	Number of Flow Deficits			Average Flow Deficit Size		
Binarization threshold	PLEX Elite	Cirrus HD-5000	Optovue Avanti	PLEX Elite	Cirrus HD-5000	Optovue Avanti
Global Default	0.39	0.41	0.82	0.29	0.46	0.85
Global Huang	0.29	0.39	0.67	0.24	0.50	0.65
Global IsoData	0.18	0.34	0.82	0.13	0.40	0.85
Global Mean	0.18	0.35	0.66	0.10	0.39	0.72
Global Otsu	0.27	0.41	0.79	0.25	0.47	0.83
Local Bernsen	0.42	0.36	0.78	0.29	0.35	0.74
Local Mean	0.41	0.34	0.72	0.35	0.40	0.79
Local Median	0.17	0.06	0.39	0.14	0.12	0.37
Local Niblack	0.39	0.21	0.59	0.40	0.35	0.66
Local Otsu	0.47	0.38	0.79	0.33	0.40	0.84
Local Phansalkar	0.56	0.38	0.84	0.44	0.22	0.87

### Registration, histogram normalization, and CLAHE

**Figure 1:**
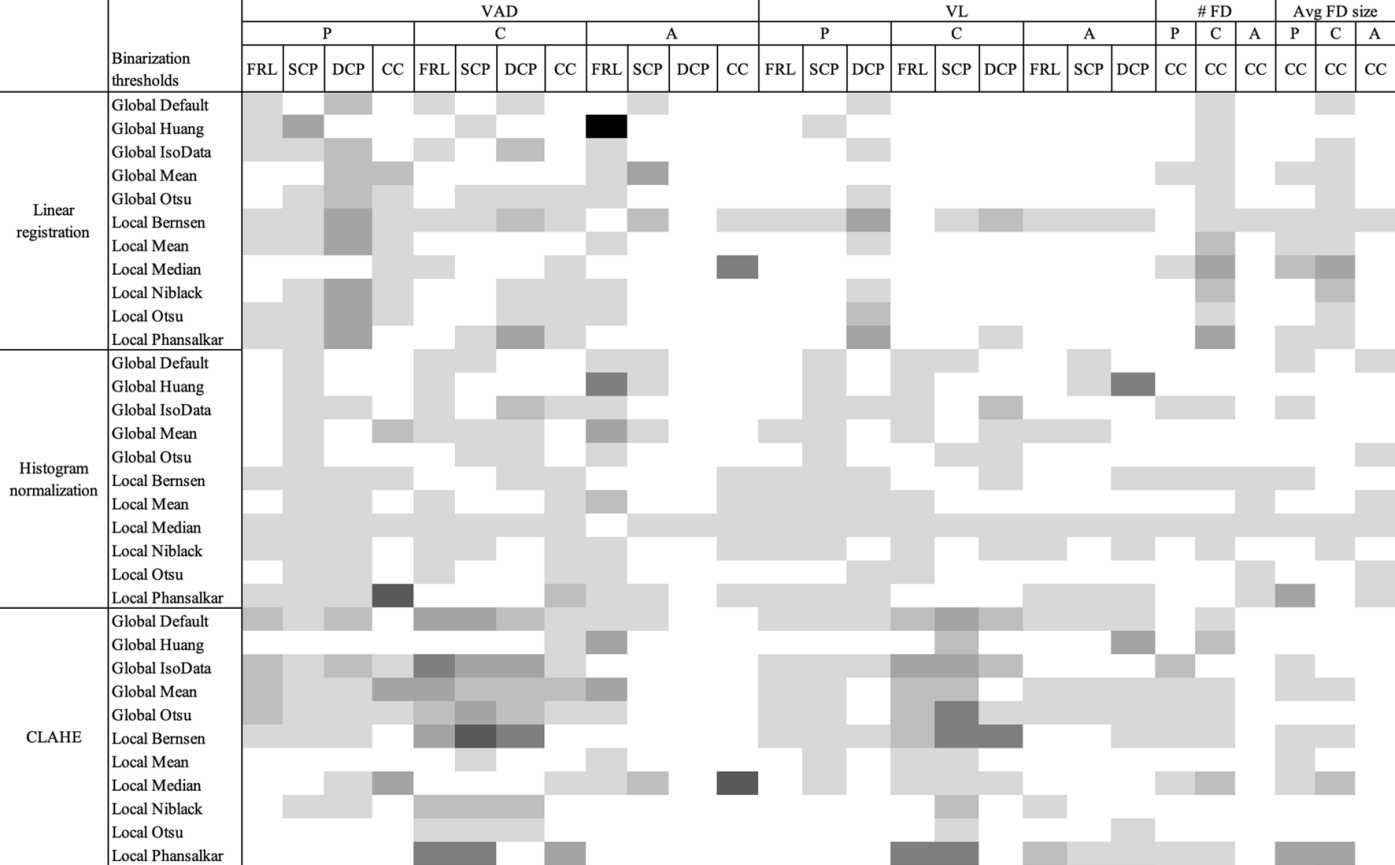
Visualization of differences in repeatability following different image processing techniques for all metrics across binarization thresholds, devices, and plexuses (calculated as ICC on processed image minus ICC on unprocessed image). Black boxes represent most improvement (greater than 0.5). White boxes represent no change or worsening of repeatability, lightest gray boxes represent improvement of 0–0.1. Each gray shade thereafter represents an additional decile of improvement. VAD = vessel area density, VL = vessel length, # FD = number of flow deficits, avg FD size = average flow deficit size, P = PLEX, C = Cirrus, A = Avanti, FRL = full retinal layer; SCP = superficial capillary plexus; DCP = deep capillary plexus; CC = choriocapillaris.

The differences in ICC calculations for quantitative metrics across multiple acquisitions of the same eye on the same device following linear image registration, histogram normalization, and CLAHE are summarized in Fig. 1. For all metrics, image registration did not generally result in high repeatability as measured by ICC (ICC > 0.80) with the exception of PLEX DCP images using the global default, IsoData, mean, and Otsu methods. In cases where ICCs did improve, the change was small (increase in ICC of less than 0.25) or the baseline ICC was already low. Histogram normalization did not result in high ICCs across all metrics, except for VAD in PLEX CC images using the local Phansalkar threshold (ICC improved from 0.39 to 0.83). Lastly, CLAHE similarly did not result in high ICC values for all metrics, with the exception of VAD in PLEX FRL images using the global default, IsoData, mean, and Otsu methods.

### Repeatability across contrast changes

The ICCs for quantification of the same image across contrast changes are summarized in Tables 4, 5 and 6. ICC values were generally low. Notably, VAD repeatability across contrast changes from CC images as measured by ICC was higher than from retinal vascular images. On the PLEX CC images, local thresholds showed very high ICC values with the exception of the local Bernsen and local Phansalkar methods. Trends for flow deficit quantification were similar (Table 6).

**Table 4:** Intraclass correlation coefficients for vessel area density on different devices and retinal plexuses across contrast changes.

	Device											
	PLEX Elite				Cirrus HD-5000				Optovue Avanti			
Binarization threshold	FRL	SCP	DCP	CC	FRL	SCP	DCP	CC	FRL	SCP	DCP	CC
Global Default	0.06	0.06	0.09	0.68	0.04	0.03	0.03	0.24	0.13	0.09	0.07	0.44
Global Huang	0.10	0.09	0.26	0.25	0.05	0.04	0.05	0.08	0.15	0.11	0.11	0.15
Global IsoData	0.06	0.08	0.10	0.60	0.05	0.03	0.03	0.23	0.13	0.10	0.07	0.42
Global Mean	0.06	0.07	0.08	0.59	0.04	0.02	0.03	0.21	0.07	0.06	0.04	0.44
Global Otsu	0.07	0.07	0.10	0.83	0.05	0.03	0.03	0.27	0.14	0.10	0.10	0.45
Local Bernsen	0.05	0.09	0.12	0.21	0.02	0.01	0.01	0.07	0.22	0.14	0.33	0.68
Local Mean	0.05	0.04	0.05	0.93	0.03	0.02	0.02	0.13	0.06	0.04	0.05	0.44
Local Median	0.04	0.04	0.05	1.00	0.03	0.02	0.03	0.39	0.02	0.01	0.08	1.00
Local Niblack	0.06	0.05	0.05	0.98	0.04	0.02	0.03	0.21	0.09	0.05	0.08	0.57
Local Otsu	0.06	0.05	0.06	0.91	0.04	0.02	0.02	0.19	0.12	0.07	0.09	0.45
Local Phansalkar	0.07	0.07	0.09	0.04	0.04	0.03	0.03	0.04	0.08	0.06	0.06	0.18

*FRL* full retinal layer, *SCP* superficial capillary plexus, *DCP* deep capillary plexus, *CC* choriocapillaris.

**Table 5:** Intraclass correlation coefficients for vessel length on different devices and retinal plexuses across contrast changes.

	Device								
	PLEX Elite			Cirrus HD-5000			Optovue Avanti		
Binarization threshold	FRL	SCP	DCP	FRL	SCP	DCP	FRL	SCP	DCP
Global Default	0.11	0.11	0.15	0.06	0.04	0.04	0.19	0.15	0.09
Global Huang	0.29	0.33	0.38	0.08	0.05	0.07	0.33	0.24	0.23
Global IsoData	0.11	0.13	0.16	0.07	0.04	0.04	0.19	0.15	0.10
Global Mean	0.17	0.19	0.16	0.07	0.05	0.05	0.20	0.17	0.10
Global Otsu	0.11	0.12	0.16	0.06	0.05	0.04	0.21	0.16	0.12
Local Bernsen	0.07	0.11	0.13	0.02	0.02	0.02	0.24	0.16	0.35
Local Mean	0.12	0.11	0.05	0.06	0.04	0.03	0.19	0.14	0.11
Local Median	0.21	0.17	0.04	0.09	0.05	0.03	0.54	0.27	0.47
Local Niblack	0.14	0.12	0.06	0.08	0.05	0.04	0.25	0.17	0.13
Local Otsu	0.10	0.09	0.07	0.06	0.04	0.03	0.19	0.13	0.11
Local Phansalkar	0.13	0.13	0.11	0.05	0.05	0.04	0.14	0.12	0.09

*FRL* full retinal layer, *SCP* superficial capillary plexus, *DCP* deep capillary plexus.

**Table 6:** Intraclass correlation coefficients for number and average size of flow deficits on different devices and retinal plexuses across contrast changes.

	Number of Flow Deficits			Average Flow Deficit Size		
Binarization threshold	PLEX Elite	Cirrus HD-5000	Optovue Avanti	PLEX Elite	Cirrus HD-5000	Optovue Avanti
Global Default	0.78	0.29	0.39	0.78	0.20	0.47
Global Huang	0.42	0.20	0.16	0.44	0.23	0.20
Global IsoData	0.68	0.27	0.37	0.68	0.19	0.45
Global Mean	0.74	0.42	0.49	0.72	0.31	0.48
Global Otsu	0.86	0.33	0.41	0.87	0.23	0.49
Local Bernsen	0.27	0.08	0.60	0.20	0.03	0.71
Local Mean	0.97	0.32	0.53	0.97	0.21	0.52
Local Median	1.00	0.98	1.00	1.00	0.96	1.00
Local Niblack	0.98	0.52	0.77	0.98	0.40	0.70
Local Otsu	0.94	0.29	0.42	0.94	0.18	0.49
Local Phansalkar	0.02	0.02	0.16	0.04	0.04	0.23

## Discussion

This study sought to measure the repeatability of quantification metrics generated by various methods of binarization thresholding applied in past studies, across multiple OCTA scans of the same eye and across variations in contrast on the same image. It is important to note that these repeatability measures are the product of the imaging itself and the quantification methodology, and thus reflect variation in both these steps. Although many studies have previously assessed the repeatability of OCTA metrics, these studies generally relied upon in-built quantification^1,2,34–36^. In most cases, a relatively high degree of repeatability has been shown for SCP quantification across several metrics, though VAD most consistently. Interestingly, in two studies that examined metrics across multiple devices, reproducibility was found to be poor, suggesting that quantification is not consistent between devices and that variation in the method (in this case, due to instrumentation) of quantification can significantly impair consistency^37,38^. However, as with most OCTA studies, both of these analyses chose different thresholding methods, making these results difficult to generalize to studies that apply different binarization thresholds. We applied quantification methods as they have been used in past OCTA studies. Consequently, we used a local threshold radius of 15 pixels on all devices, as has been done in past OCTA CC studies on the same three devices employed in this study. However, a 15 pixel radius corresponds to different radii sizes in microns on different devices due to variable image resolutions^25–28^. Chu et al. have undertaken an in-depth analysis of the effect of thresholding methods, including local threshold radius, and suggest that this parameter needs to be more carefully assessed and optimized in future OCTA studies that employ local thresholding methods^5^.

We assessed repeatability using the ICC, a commonly applied statistic which accounts for variation within and between subjects. Because of this, the ICC places repeatability in the context of overall variation in the data set. This is a useful property but also one that needs to be considered in interpreting ICCs. In the context of this study of all normal eyes, it means that the relatively low variation between subjects may have decreased ICCs and that the measured repeatability could be greater in a more heterogenous data set, such as one incorporating pathological eyes. While coefficient of repeatability, another common statistic which gives a “confidence interval” for measurement uncertainty, is likely more clinically useful, it does not account for overall variation in the data set and thus we have reported ICC. Future studies should measure and establish coefficients of repeatability for commonly applied methods. Finally, it is important to note that repeatability measures should not be interpreted without validation in terms of accuracy. A binarization method that consistently turns all images entirely white, for example, would achieve “perfect” repeatability as measured by coefficient of variation and, potentially, ICC (depending on the between-subject variation) but would be completely inaccurate. This underscores that repeatability alone is inadequate in assessing a methodology, and that a gold standard for OCTA metric validation is still needed. Such a gold standard will likely rely upon, at least initially, established techniques such as histopathology, color fundus photography or adaptive optics, against which measurements of vasculature on OCTA imaging can be compared. It should also be considered whether different gold standards may be needed depending on the layer being studied—the choriocapillaris versus the retinal vasculature, for example.

To our knowledge only two prior studies have compared the repeatability of various binarization thresholding methods in quantification of OCTA images^5,39^. Shoji et al. examined the repeatability of macular VAD from PLEX and Topcon Triton SS-OCTA images of the SCP between six binarization thresholding methods. They also found inconsistent repeatability, with ICCs ranging from 0.22 to 0.88 depending on the thresholding method and device used for SCP VAD quantification. More recently, Chu et al. performed an in-depth analysis, including assessing repeatability, of two binarization methods for CC analyses, including the Phansalkar local threshold, and found higher repeatability than in the present study. Notably, Chu et al. used a different segmentation strategy for the CC that may have contributed to improved repeatability, suggesting that future studies should carefully consider their segmentation boundaries instead of relying on the default settings. In many ways, the present study builds on these studies in that we have analyzed images from different plexuses across three devices using a relatively comprehensive list of binarization thresholding algorithms that have been applied in past studies. Moreover, we have examined the effects of several image processing techniques on repeatability, a critical element as these adjustments are often incorporated into image analysis^19,26,40^ and can have a synergistic effect with binarization thresholding methods in increasing the variation of resulting quantification metrics^3^. Our findings suggest that repeatability as measured by ICC was inconsistent and, for most binarization methods, devices, plexuses, and metrics, relatively low. Although we employed a cut-off of 0.8 for “high” repeatability, it has been argued that a threshold of at least 0.9 is more appropriate^41^. This may be particularly true in medical applications if clinical determinations are made from the measured data.

**Figure 2:**
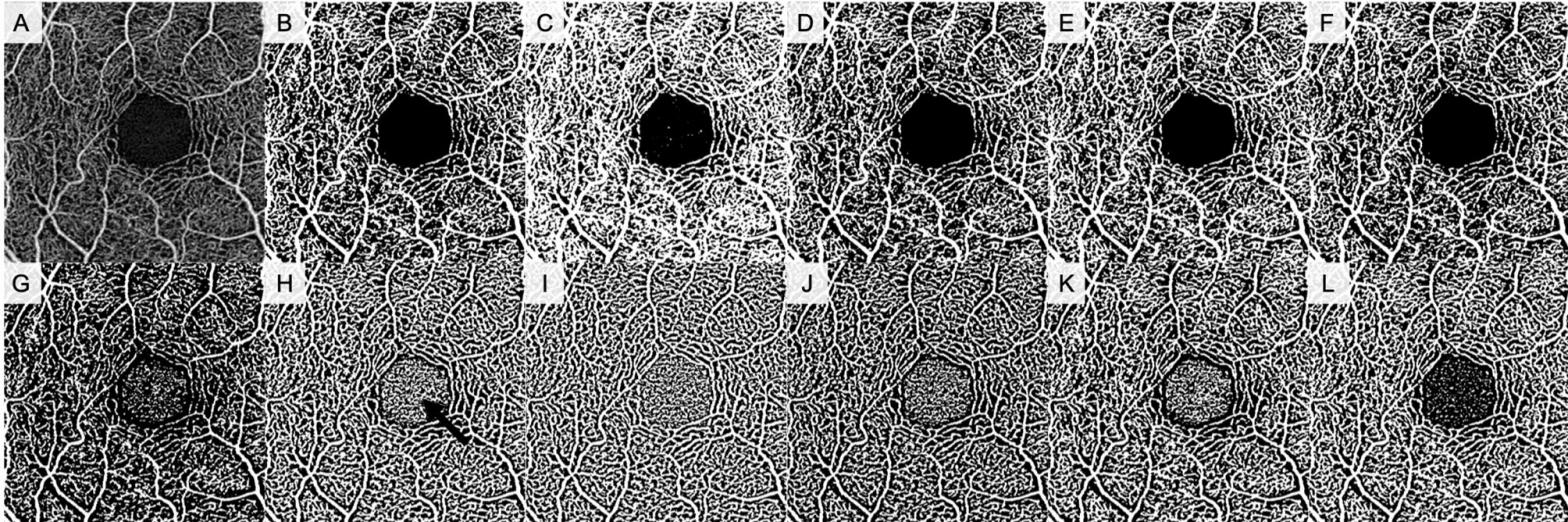
Superficial capillary plexus images binarized using various algorithms. Top row: (**A**) Original unbinarized image, (**B**) global default, (**C**) global Huang, (**D**) global IsoData, (**E**) global mean, (**F**) global Otsu. Bottom row: (**G**) local Bernsen, (**H**) local mean, (**I**) local median, (**J**) local Niblack, (**K**) local Otsu, (**L**) local Phansalkar. Black arrow in image H indicates significant FAZ noise introduced by most local thresholding methods.

In the absence of a true gold standard for validation of various methods, the question of which method is “best” cannot be answered. However, using known avascular areas such as the foveal avascular zone (FAZ) to assess accuracy is a useful comparison. Local thresholds tend to erroneously binarize the FAZ as white, producing incorrect binarized images (Fig. 2)—a point we have made previously^3^. Qualitatively, the local Phansalkar threshold seems to produce the least over-segmentation in large avascular areas among local binarization thresholds, and so may be appropriate for use in certain situations, such as in studies that seek to detect the effects of small contrast differences. Increasing the radius size of the local thresholding algorithm, as undertaken by Chu et al.^5^ may also mitigate this issue, although we did not examine this. Thus, regardless of repeatability, for any quantification of retinal plexus OCTA images, local thresholds should only be used if the FAZ is excluded from analysis. Different thresholding methods may therefore be appropriate for different analysis situations. It is important to note that the use of local thresholding methods is largely predicated on the assumption that compensation for image heterogeneity is necessary or advantageous^25^; ideally, if OCTA data are adequately adaptively normalized, global thresholding methods would be sufficient. Notably, the Avanti Optovue SD-OCTA consistently showed the highest repeatability in CC quantification, even more so than the PLEX Elite SS-OCTA. This may be due to differences in image acquisition between the two systems; the Avanti Optovue utilizes merging of a *x-* and *y*-fast scan, which may contribute to increased quantification repeatability. Further investigation is needed to better assess this question.

**Figure 3:**
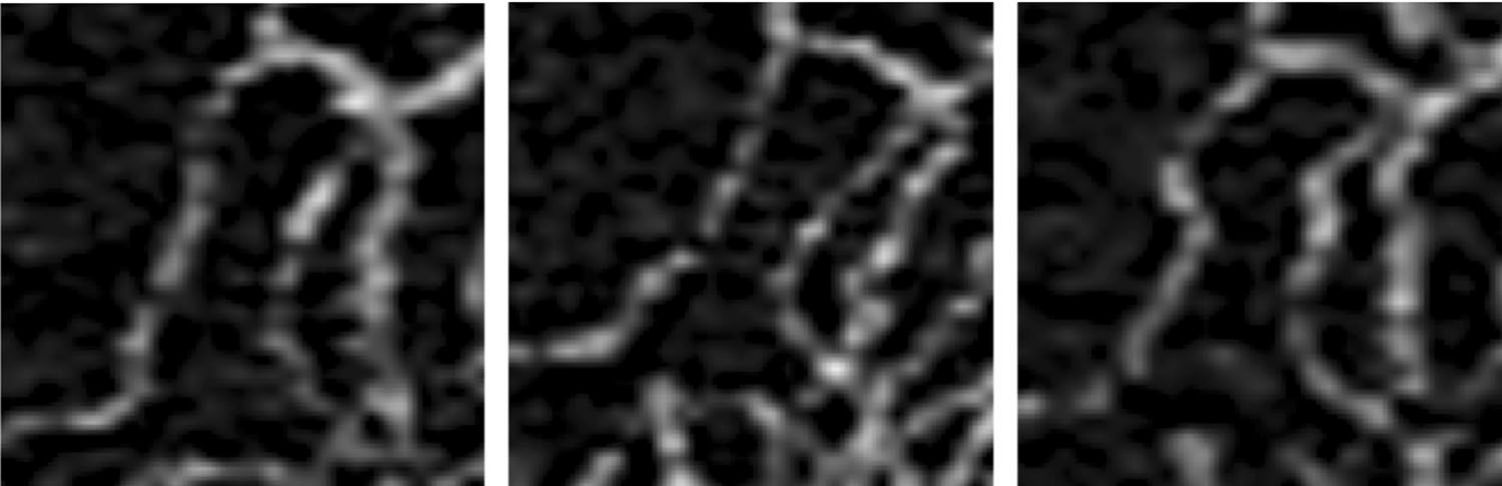
Magnified images of the same vessel segment in three repeated full retinal layer en-face OCTA scans of the same eye. There is clear variation in vessel appearance, including shape, caliber, and continuity, which may inherently limit repeatability.

This study also assessed whether linear image registration, histogram normalization, and CLAHE improve repeatability. None of these changes resulted in consistent improvements in the ICC values for any thresholding method (Fig. 1). Image registration accounts for small misalignment between images, due, for example, to variations in fixation. Histogram normalization and CLAHE are different algorithmic approaches that attempt to “optimize” contrast (i.e., maximize without over-contrasting) between images by ensuring the distribution of pixel values is consistently 0–255 for all images. The fact that none of these methods resulted in high repeatability suggests that other intrinsic interscan differences (apart from changes in alignment or image contrast) are primarily driving variation. Small differences in vessel visualization due to variations in eye motion, tracking, and image focusing—which could be due to dynamic changes in blood flow^42^—cannot be accounted for in post-processing and could have a major impact on the repeatability of quantification (Fig. 3). Scanning parameters such as interscan time and the number of repeated scans can also impact the degree of variation between scans: An increase in interscan time and the number of repeated B-scans would decrease variability between scans, but longer interscan times can increase artifact and noise, and both strategies require longer acquisitions^43,44^. In addition, small differences in the automatically detected segmentation boundaries could affect vessel visualization in the en face scans.

**Figure 4:**
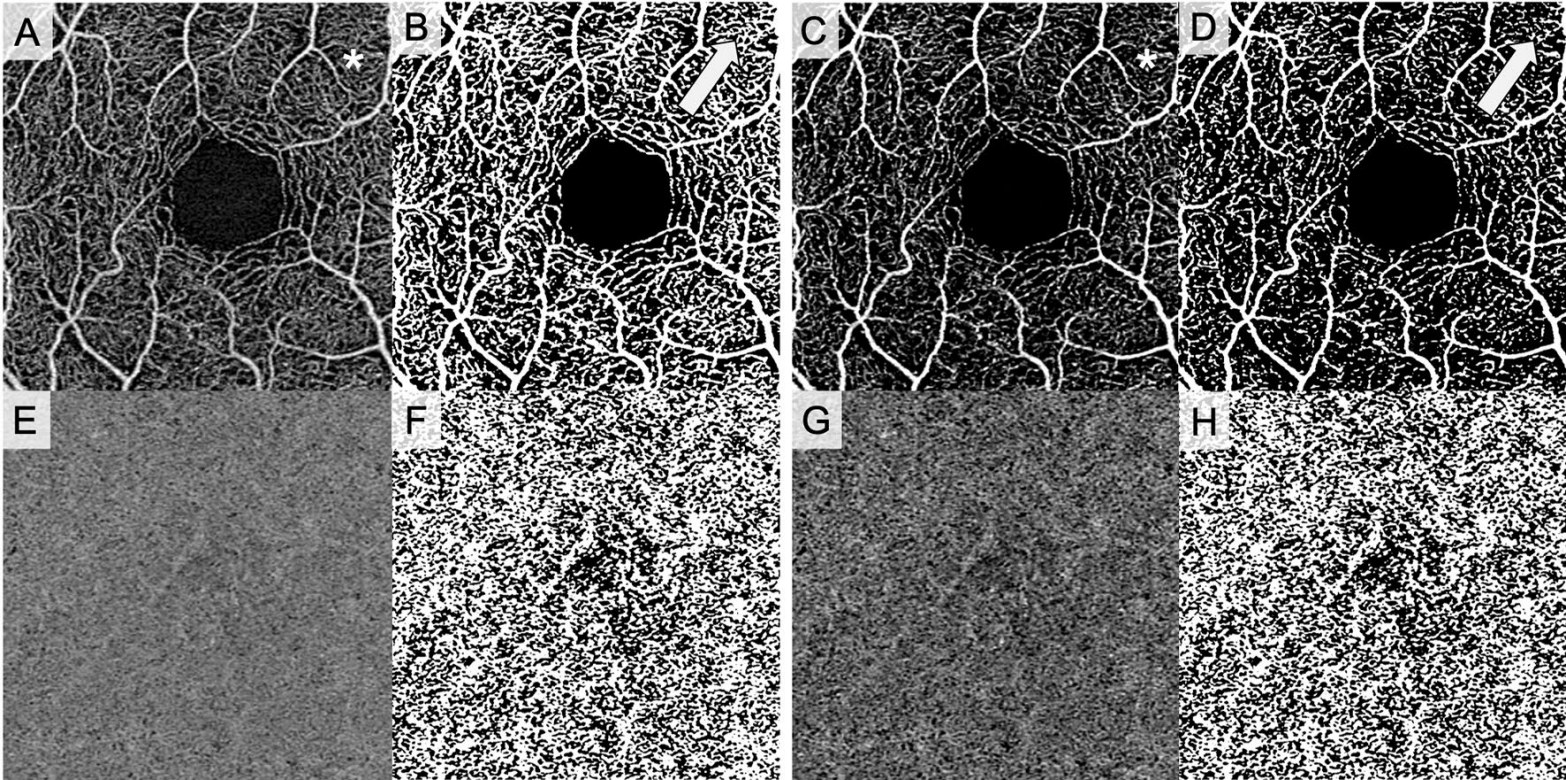
Effect of contrast change on SCP and CC original and binarized images. (**A**) Original unaltered SCP image, (**B**) binarized unaltered SCP image, (**C**) contrast-increased SCP image, (**D**) contrast-increased binarized SCP image, (**E**) original unaltered CC image, (**F**) binarized unaltered CC image, (**G**) contrast-increased CC image, (**H**) binarized contrast-increased CC image. Binarized images in this figure were produced using the global Otsu threshold for illustration purposes. Asterisks indicate areas of noticeable small vessel loss between original and contrast-increased SCP images. Arrows indicate same areas in unaltered and contrast-increased binarized SCP images.

Finally, we assessed the repeatability of various binarization thresholds when contrast changes are made on the same image (Tables 4, 5, 6). This is a critical question; many OCTA systems allow the operator to change the image contrast even before exporting, and some research studies have made contrast adjustments during analysis for a variety of reasons, include to optimize image appearance^19,26,40^. For VAD, we found that there was poor repeatability in retinal vascular quantification on all devices, with ICCs ranging from 0.01 to 0.32 (Table 4). Interestingly, CC quantification was considerably more repeatable across contrast changes, particularly using local thresholds. On the PLEX Elite, for example, the local Niblack and local Otsu thresholds showed ICC values of 0.98 and 0.91, respectively, across contrast changes. The global Otsu also showed high repeatability on the PLEX Elite (ICC 0.83). The notable exception was the local Phansalkar threshold, which has been primarily used in recent CC studies^25–28^. The local Phansalkar method showed very low repeatability across contrast changes—this is not surprising, giving that this algorithm was designed for low contrast images and thus is highly susceptible to small contrast alterations^3,45^. The higher repeatability of CC versus retinal vascular quantification may initially seem surprising, given the difficulty of imaging the CC accurately. However, inspection of the effect of increased contrast on SCP versus CC images provides some clarity. As is evident in Fig. 4, increasing contrast in a SCP image (image A to C) results in noticeable loss of small vessels. However, while increasing contrast in a CC image (image E to G) does make flow deficits more visible, this effect is consistent across the image. In other words, normal retinal vascular macular OCTA images are relatively heterogenous, containing large vessels, small vessels and capillaries, intercapillary flow deficits, and the FAZ. Normal CC images, on the other hand, are more homogenous, containing flow areas and flow deficits distributed fairly consistently across the imaging area. Contrast changes thus can affect the various components of retinal vascular images differently, variations that are maintained or even accentuated during the binarization process and that then translate into altered metrics; note the difference between binarized image B (no contrast change) versus D (increased contrast). Any global adjustment made to a CC image, however, will affect the entire image relatively consistently (images F versus H). This, of course, may not be the case in images of pathologic eyes, as numerous diseases can cause CC loss that will result in more heterogenous images and likely greater difficulty in accurate binarization^27,46,47^. If not carefully applied, image contrast changes, such as those that can be made on devices themselves, can result in clipping of OCTA signal by setting low or high intensity pixel values to 0. This is likely part of the reason our analysis shows low repeatability across contrast changes in the retinal plexus images, and these results underscore that contrast changes need to be applied carefully in image analysis.

Overall, these results suggest that the repeatability of various methods is inconsistent. It is important to note that low repeatability in and of itself does not imply a particular method should not be used; without a gold standard to validate quantification metrics, such a determination cannot be properly made in the context of a research study. Instead, our study underscores the need for a ground truth against which metrics can be compared. In the absence of such a ground truth, it stresses the importance of the development of some common standards across different studies and especially in the conduct of clinical trials. We have previously shown that inconsistency in methods can even influence the directionality of trends in comparative studies, not just the absolute value of quantitative metrics^3^.

There were several limitations to this study. First, only normal eyes were examined. Our group has initially assessed the repeatability of OCTA measurements in diabetic eyes in one published separate study^48^—in future, it would be valuable to complete similar analysis with manual quantification using a range of binarization techniques. In addition, three devices were used in this study. Although these are three commonly used systems, there are numerous additional OCTA devices currently in use that should be assessed in future repeatability studies. We also examined eleven binarization thresholds, but there is even greater variety in the methods used in published studies, particularly custom thresholds that are difficult to duplicate. However, we tried to create a relatively exhaustive list of commonly used methods that have been applied in prior studies. We also used the default automated segmentation for each device; several studies have suggested that these segmentation boundaries may not necessarily be accurate, particularly for the CC^5,49^. For CC quantification, we report VAD instead of flow deficit percentage (FDP) in order to facilitate comparisons with past CC studies that have also reported VAD. However, because of the limited resolutions of the OCTA systems, VAD is not an ideal OCTA metric for the CC and flow deficit percent (FDP) is likely a better option for future studies. Finally, our study only examined 3 × 3 mm foveal en-face OCTA images; there is likely different variability in other commonly used scan patterns, such as 6 × 6 mm or 12 × 12 mm.

## Conclusion

There is variable repeatability in quantification of 3 × 3 mm OCTA images of the retinal vascular plexuses and CC across a variety of devices, metrics, and binarization thresholds. Neither linear registration, histogram equalization, nor CLAHE resulted in high repeatability in most cases. No binarization thresholding method is highly repeatable in retinal vascular quantification across contrast changes, while CC quantification is more repeatable over variable contrast, particularly when using local thresholds. To be comparable, OCTA studies should employ a set of common methodologies or standards that allow interstudy comparisons.

## Supplementary information

Supplementary Figures.

## Data Availability

The data sets generated during and/or analyzed during the current study are available from the corresponding author on reasonable request.
