# Supplementary material for: Repeatability of binarization thresholding methods for optical coherence tomography angiography image quantification
Source: Sci Rep. 2020 Sep 21;10:15368. doi: 10.1038/s41598-020-72358-z (PMC7505834; doi:10.1038/s41598-020-72358-z)
Supplement: Supplementary file 1 — Supplementary Figures. [file 41598_2020_72358_MOESM1_ESM.docx]

**Repeatability of Binarization Thresholding Methods** **for Optical Coherence Tomography Angiography Image Quantification**

Nihaal Mehta MD^1, 2^, Phillip X. Braun MD^1, 3^, Isaac Gendelman MD^1^, A. Yasin Alibhai MD^1^, Malvika Arya MD^1^, Jay S. Duker MD^1^, Nadia K. Waheed MD MPH^1^

^1^ New England Eye Center, Tufts Medical Center, Boston, Massachusetts, USA

^2^ The Warren Alpert Medical School of Brown University, Providence, Rhode Island, USA

^3^ Yale School of Medicine, New Haven, Connecticut, USA


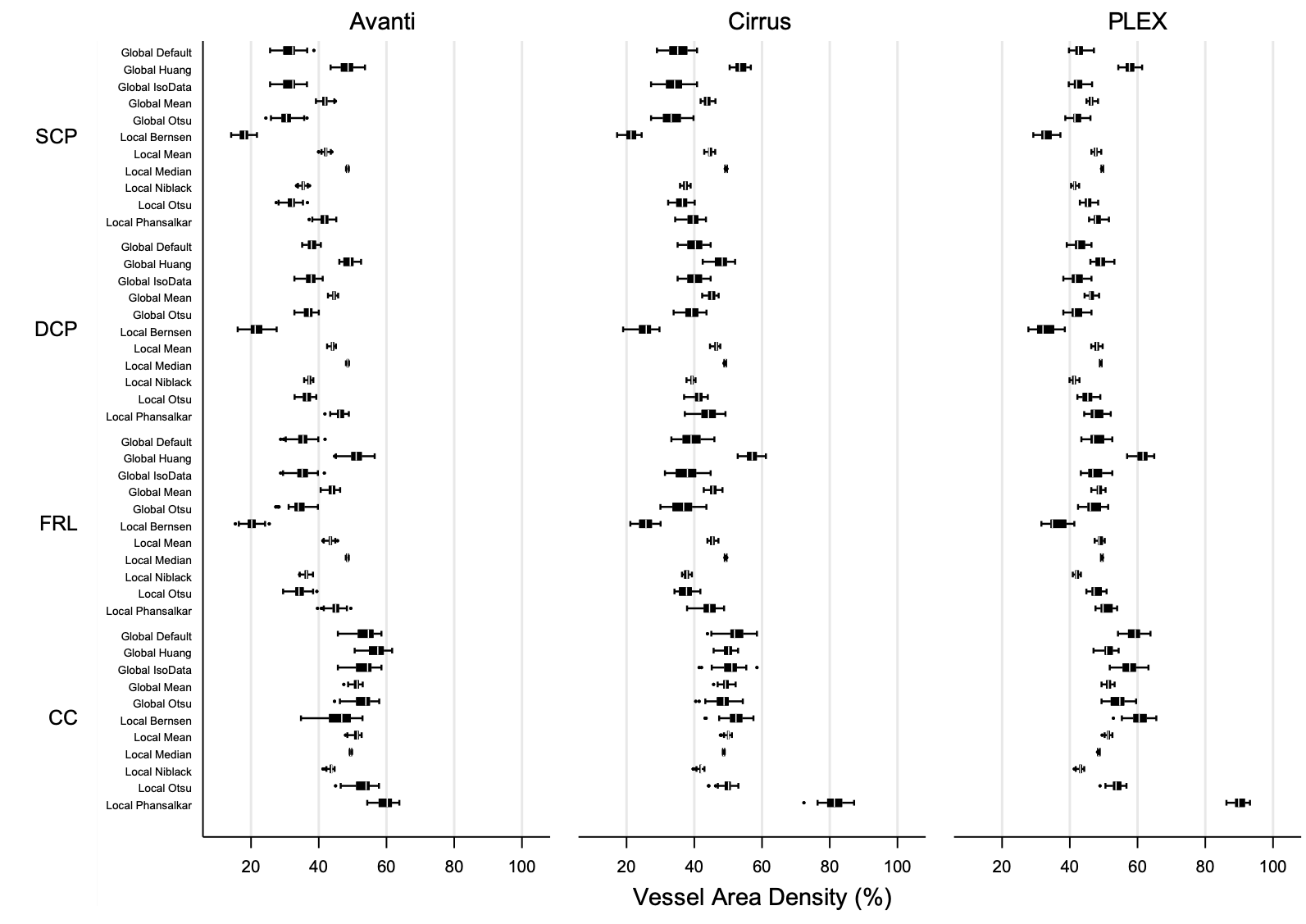


Supplementary Figure 1: Box-and-whisker plots summarizing vessel area density distribution by device, plexus, and binarization method. Boxes indicate 25^th^-75^th^ percentile, and whiskers indicate range. SCP = superficial capillary plexus, DCP = deep capillary plexus, FRL = full retinal layer, CC = choriocapillaris.


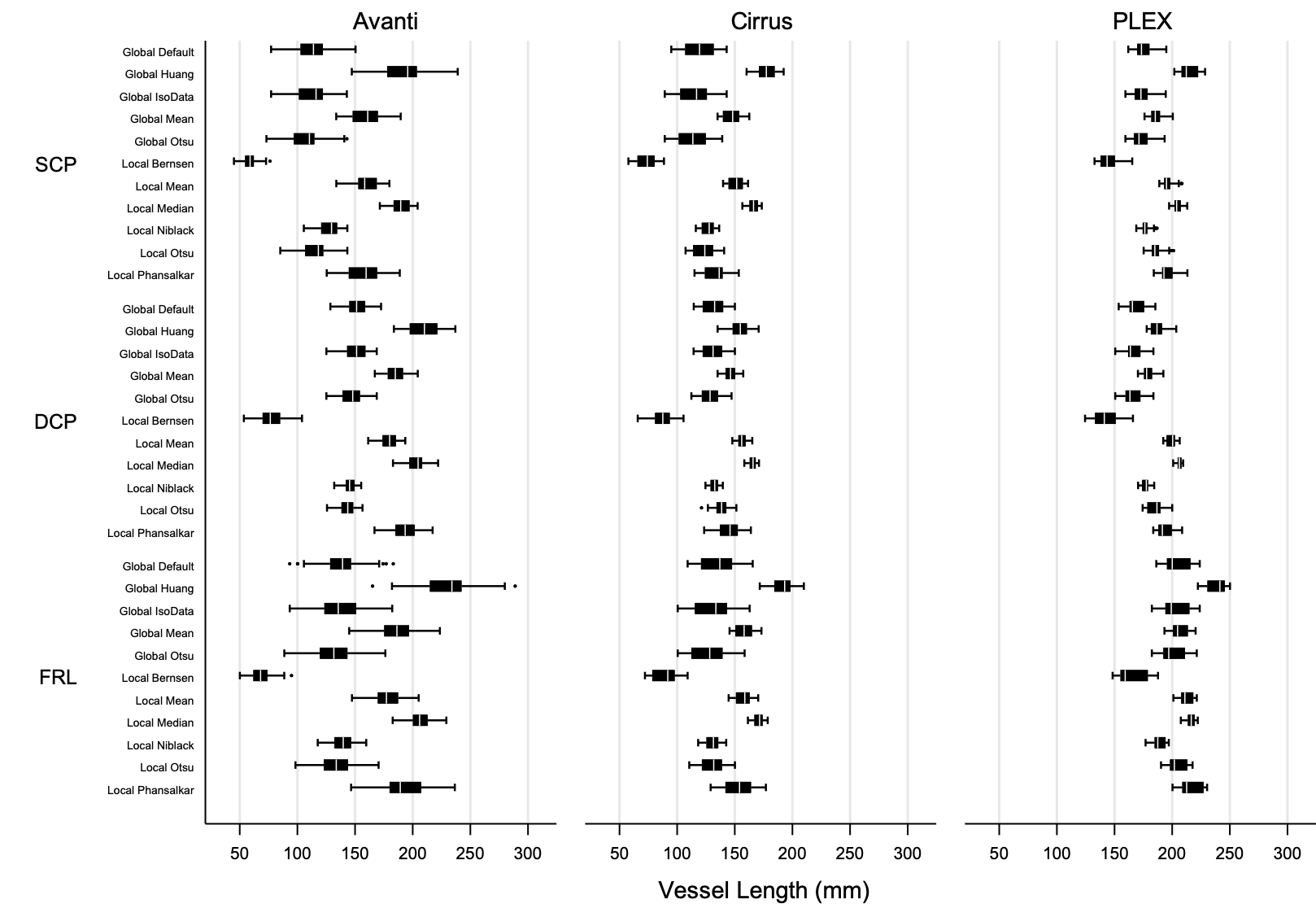
Supplementary Figure 2: Box-and-whisker plots summarizing vessel length distribution by device, plexus, and binarization method. Boxes indicate 25^th^-75^th^ percentile, and whiskers indicate range. SCP = superficial capillary plexus, DCP = deep capillary plexus, FRL = full retinal layer.


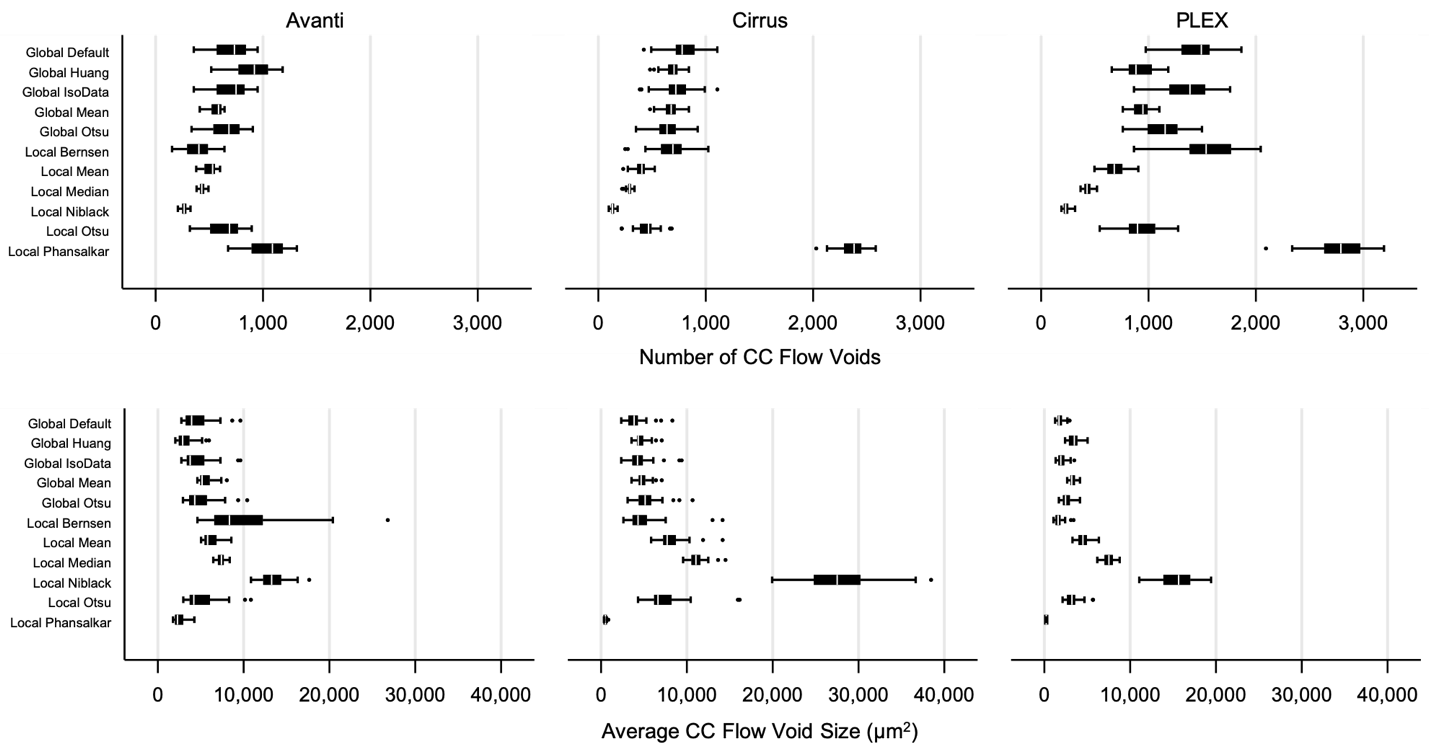
Supplementary Figure 3: Box-and-whisker plots summarizing number of choriocapillaris flow voids and average choriocapillaris flow void size distribution by device, plexus, and binarization method. Boxes indicate 25^th^-75^th^ percentile, and whiskers indicate range. CC = choriocapillaris.
